# Antioxidant and Anti-Obesity Effects of *Juglans mandshurica* in 3T3-L1 Cells and High-Fat Diet Obese Rats

**DOI:** 10.4014/jmb.2311.11032

**Published:** 2023-12-18

**Authors:** Da-Hye Choi, Min Hong, Tae-Hyung Kwon, Soo-Ung Lee

**Affiliations:** Institute of Biological Resources, Chuncheon Bioindustry Foundation, Chuncheon 24232, Republic of Korea

**Keywords:** Anti-obesity, antioxidant, adipogenesis, lipogenesis, *Juglans mandshurica* Maxim

## Abstract

*Juglans mandshurica* Maxim. walnut (JMW) is well-known for the treatment of dermatosis, cancer, gastritis, diarrhea, and leukorrhea in Korea. However, the molecular mechanism underlying its anti-obesity activity remains unknown. In the current study, we aimed to determine whether JMW can influence adipogenesis in 3T3-L1 preadipocytes and high-fat diet rats and determine the antioxidant activity. The 20% ethanol extract of JMW (JMWE) had a total polyphenol content of 133.33 ± 2.60 mg GAE/g. Considering the antioxidant capacity, the ABTS and DPPH values of 200 μg/ml of JMWE were 95.69 ± 0.94 and 79.38 ± 1.55%, respectively. To assess the anti-obesity activity of JMWE, we analyzed the cell viability, fat accumulation, and adipogenesis-related factors, including CCAAT-enhancer-binding protein alpha (C/EBPα), sterol regulatory element-binding protein-1c (SREBP1c), peroxisome proliferator-activated receptor-gamma (PPARγ), fatty acid synthase (FAS), and acetyl-CoA carboxylase (ACC). We found that total lipid accumulation and triglyceride levels were reduced, and the fat accumulation rate decreased in a dose-dependent manner. Furthermore, JMWE suppressed adipogenesis-related factors C/EBPα, PPARγ, and SREBP1c, as well as FAS and ACC, both related to lipogenesis. Moreover, animal experiments revealed that JMWE could be employed to prevent and treat obesity-related diseases. Hence, JMWE could be developed as a healthy functional food and further explored as an anti-obesity drug.

## Introduction

Obesity is a rapidly growing global health challenge that markedly increases the risk of chronic diseases such as hyperlipidemia, hypertension, and diabetes. Typically, obesity can be attributed to an imbalance between fat synthesis and degradation, causing various metabolic diseases that adversely affect the health of individuals. It is further characterized by the differentiation of undifferentiated preadipocytes into mature adipocytes, a process termed adipogenesis [1], which involves the accumulation of triglycerides (TGs) and lipid droplets within adipocytes, thereby expanding adipose cells and resulting in increased insulin resistance [2, 3].

Various transcription factors are known to be involved in adipocyte differentiation. Typical transcription factors include sterol regulatory element-binding protein-1c (SREBP1c), peroxisome proliferator-activator receptor-gamma (PPARγ), and CCAAT/enhancer binding protein-α (C/EBPα); these are considered representative regulators of adipogenesis [4]. SREBP1c promotes the expression of various transcription factors. Moreover, several lipid droplet-producing enzyme products, including C/EBPα and PPARγ, participate in the early stages of lipid differentiation, whereas fatty acid synthase (FAS) and acetyl-CoA carboxylase (ACC) are involved in the late stage of lipid droplet differentiation [4, 5]. Lipogenic enzymes induce the tissue uptake of plasma TGs, as well as the conversion of acetyl-CoA into fatty acids and TGs [6].

Lipogenesis primarily occurs in the cytoplasm of mature adipocytes and is mediated by acetyl-CoA. ACC converts acetyl-CoA to malonyl-CoA, while the FAS complex mediates fatty acid synthesis. Fatty acids are subsequently produced from fatty acyl-CoA and combined with glycerol to synthesize TGs [7]. Fatty acid binding protein 4 plays a key role in fatty acid transmembrane transport. Typically, TG production occurs via the ionization of fatty acids into glycerol [8]. Therefore, it is necessary to suppress adipogenesis and lipogenesis in the adipocytes to reduce TG accumulation. The phosphorylation of AMP-activated protein kinases can occur via various pathways. For example, the activation of the β-oxidation of fatty acids by suppressing SREBP1-c and inhibition of lipid synthesis pathways such as FAS [9-11]. Accordingly, this lipid metabolism process indicates that the regulation of adipogenesis can help control obesity.

Recent studies have highlighted the adverse side effects associated with approved anti-obesity drugs and bariatric surgeries [12]. Therefore, dieting has emerged as an alternative for obesity control, emphasizing the consumption of healthy foods such as nuts.

The deciduous tree *Juglans mandshurica* Maxim, a member of the Juglandaceae family, is widely distributed in northeastern China and Korea. Traditional medicine has long recognized the potential of walnuts from *J. mandshurica* Maxim., generally known as the *J. mandshurica* walnut (JMW), for the treatment of dermatosis, cancer, gastritis, diarrhea, and leukorrhea in Korea [13, 14]. Furthermore, individuals with hyperlipidemia who consumed 20 g/day of J. regia L. exhibited a notable reduction in their TG levels of approximately 17%, along with a marked increase in their plasma high-density lipoprotein (HDL)-cholesterol concentrations [2]. *J. mandshurica* Matrix can be divided into various parts, such as leaves, branches, and fruits. In particular, the fruit, denoted as JMW, contains various active ingredients, such as ellagic acid [15], juglanin [16-18], and juglone [19], well-recognized to exert antitumor, anti-inflammation, antioxidative, anti-microbial activities. However, the anti-obesity potential of the active ingredients of JMW remains poorly explored. Therefore, we aimed to establish whether JMW could regulate obesity in 3T3-L1 and high-fat diet obese rats

## Materials and Methods

### Sample Preparation

*J. mandshurica* Maxim. walnut (JMW) fruit originating from Chuncheon, South Korea, was purchased from the Internet. The fruits were ground using a DA380-S blender (Daesung Artlon Co., Ltd., Republic of Korea), and the contents were extracted with 20% ethanol. The extract was then filtered, evaporated, and lyophilized in a freeze dryer (ilShinBioBase Co., Ltd., Republic of Korea). The obtained powder was dissolved in a 10% dimethyl sulfoxide (DMSO) solvent and used for subsequent experiments. To obtain the JMW ethanol extract (JMWE), the 20% ethanol extract was shaken at 150 rpm for 6 h at 25°C.

### Reagents

The antioxidant activity of JMWE was assessed using 2,2'-Azino-bis-3-ethylbenzthiazoline-6-sulfonic acid (ABTS), which was obtained from Wako Pure Chemical Industries, Ltd. (Japan). Furthermore, Sigma-Aldrich (USA) provided the Folin-Ciocalteu phenol reagent, potassium ferricyanide, dimethyl sulfoxide, trichloroacetic acid, ferric chloride, butylated hydroxyanisole, and ascorbic acid (AA).

### Total Polyphenol Content Assay

The Folin-Denis method [20], with modifications, was used to measure the total polyphenol content (TPC) of JMWE. Briefly, JMWE and the Folin-Denis phenol reagent were combined in a 1:1 ratio and allowed to react for 3 min. Following the addition of 10% Na_2_CO_3_, the samples were incubated at 25°C for 1 h. A SpectraMax M5 plate reader (Molecular Devices, USA) was used to measure the absorbance of the reactants at 760 nm. The TPC of JMWE is stated in milligrams of gallic acid equivalents (GAE) per gram. Each sample was examined in triplicate.

### DPPH and ABTS Activity Assays

The procedure for performing the 1,1-diphenyl-2-picrylhydrazyl (DPPH) radical scavenging experiment was first described by Blois (1958). Briefly, a 1:2 reaction between JMWE and 0.2 mM DPPH solution was initiated by vortexing at 25°C for 10 min. Using a SpectraMax M5 plate reader, the absorbance of reactants was determined at 517 nm. Each experiment was performed in triplicate. Utilizing the protocol outlined by Re *et al*. [21], we performed the ABTS radical cation decolorization assay. To summarize, 2.45 mM potassium persulfate and 7 mM ABTS were mixed together, and the mixture was left to incubate for 24 h at 25°C in the dark. JMWE and the ABTS solution were mixed together, allowed to sit at room temperature for ten minutes, and then stirred once more. At a wavelength of 734 nm, the reactant absorbance was computed. The antioxidant activity was calculated using the following formula:

Scavenging effect (%) = [1 − A_(S)_ − A_(SB)_ / A_(C)_] × 100

S, Sample; SB, Sample black; C, Control.

### Reducing Power

The reducing power of JMWE was determined according to the Oyaizu method [22]. A reaction mixture containing 1 ml of JMWE and 1 ml of 1% potassium ferricyanide was incubated in a water bath at 50°C for 20 min. After incubation, 10% trichloroacetic acid was added, centrifuged at 3,000 ×*g* for 10 min. Next, 1 ml of upper layer was added to 1 ml distilled water and 0.1 ml of 0.1% ferric chloride. The solution was quantified at 700 nm. Each sample assay was performed in triplicate. The results were compared with BHA and AA.

### Anti-Obesity Assessment

The 3T3-L1 cell differentiation process was assessed using fetal bovine serum (FBS; Gibco) and Dulbeccós modified Eaglés medium (DMEM, Sigma-Aldrich, USA). Reagents used in the first experiment stage were dexamethasone (DEX), 3-isobutyl-1-methylxanthine (IBMX), and insulin (INS), all purchased from Sigma-Aldrich. The presence of ellagic acid in the JMWE powder was confirmed using reagents procured from Sigma-Aldrich.

### Cell Counting Kit-8 (CCK-8) Assay

Following JMWE treatment, cell viability was measured using the CCK-8 assay (Dojindo, Japan). CCK-8 was used to assess the cell proliferation and viability of JMWE-treated 3T3-L1. Briefly, 3T3-L1 cells at a density of 1 × 10^5^ cells/well were cultured in a 96-well plate (SPL Life Science, Republic of Korea). The culture was incubated under a 5% atmosphere of CO_2_ at 37°C. To evaluate cell viability, we used various concentrations of JMWE, including 50, 100, and 200 μg/ml. Absorbance was measured at 450 nm.

### In Vitro 3T3-L1 Cell Differentiation Analysis

The 3T3-L1 preadipocytes were cultured in T-flasks using DMEM containing 10% bovine calf serum at 37°C and under 5% CO_2_. Cell confluency was maintained above 70% by subculturing. On reaching 80–90% confluency, post-confluent 3T3-L1 preadipocytes (day 0) were grown in an induction medium containing DMEM with 10%(v/v) FBS for three days (from day 0 to 3). The medium contained MDI (0.5 mM 3-isobutyl-1-methylxanthine, 10 μg/ml insulin, and 1 μM DEX). The media was swapped for DMEM with 10% FBS and 20 μg/μl INS. Subsequently, the medium was replaced every two days for eight days with fresh DMEM containing 10% FBS and JMWE at concentrations of 50, 100, and 200 μg/ml.

### Oil Red O Staining

The 3T3-L1 cells were rinsed five times using phosphate-buffered saline (PBS) and fixed in 4% formaldehyde for 1 h. Once fixed, the cells were dehydrated using 60% isopropyl alcohol. Then, a 0.5% Oil Red O staining solution in 60% isopropanol was diluted with distilled water (3:2) and filtered through a bottle-top filter (Nalgene, Thermo Fisher Scientific Inc., USA). The staining solution was dropped onto the cells on the plate, followed by incubation in the dark for 10 min. Stained lipid droplets in the cells were observed under a microscope. After extracting the Oil Red O staining solution from the cells using 100% isopropanol, the absorbance of stained lipid droplets was measured at 520 nm.

### Enzymatic Method for Measuring Extracellular TGs

After differentiation (preadipocytes into adipocytes), the supernatant of the culture medium was used for TG content analysis using a Konelab 20i biochemistry analyzer (Thermo Electron Corp., Finland). The experiment was performed according to the manual instructions.

### Total RNA Isolation and Reverse Transcription-Quantitative PCR

Completely differentiated 3T3-L1 cells were rinsed three times with PBS. After rinsing, the cells were collected using cell scrapers to extract RNA. An RNA isolation kit (Qiagen, Inc., USA) was used to extract RNA with high purity according to the manufacturer’s protocol. The RNA purity was measured using a NanoDrop spectrophotometer (Thermo Fisher Scientific). One microgram of the extracted RNA was used for cDNA synthesis using the Reverse Transcription Master Premix (Elpis-Biotech, Republic of Korea). Gene expression levels were analyzed via real-time RT-qPCR using a LightCycler 480 Instrument II (Roche, Germany). Table 1 presents the primer sequences used. SYBR Mixture (Roche) was used for 45 cycles of RT-qPCR performed on a LightCycler 480 Instrument II (Roche). Gene expression was calculated using the 2^−ΔΔCt^ method. The RT-qPCR cycling conditions were as follows: 5 min at 95°C, 40 cycles of 15 s at 95°C, 15 s at 58°C, and 30 s at 72°C. For relative quantitative analysis, the protein expression level was normalized to that of β-actin, and the gene expression rate was measured by dividing the fold change value of the reference gene.

### Western Blot Analysis

Antibodies used for western blotting analysis, including PPARγ, C/EBPα, FAS, ACC, β-actin, and anti-rabbit IgG, were purchased from Cell Signaling Technology (USA). The anti-SREBP1c antibody was obtained from Abcam (USA). Following adipogenesis and lipogenesis processes, 3T3-L1 cell proteins were collected using a cell scraper at the end of the cell differentiation process. The proteins were then washed twice with PBS and extracted using a PRO-PREPTM (iNtRON Biotechnology, Republic of Korea). The precise protein concentration in cells was measured using a PierceTM BCA Protein Assay kit (Thermo Fisher Scientific). The proteins were separated using sodium dodecyl sulfate-polyacrylamide gel electrophoresis. The electrophoresis gel was then transferred to a PVDF membrane by submerging in Tris-buffered saline (TBS-T) buffer containing 5% BSA (Sigma-Aldrich) for 1 h to block nonspecific binding of antibodies. The membranes were probed with primary antibodies diluted at 1/ 500 for 24 h. After washing with TBS-T buffer three times, the membrane was incubated with TBS-T buffer containing secondary antibodies for 2 h. SuperSignalTM Western Blot Enhancer (USA) was used to detect antibody immunostaining. The ImageQuant LAS-4000 (Fujifilm, Japan) was used to analyze luminosity.

### High-Performance Liquid Chromatography (HPLC) Analysis

HPLC (Shimadzu, Japan) was performed to analyze the main compounds in JMWE. Utilizing a ProntoSIL C18-ace-EPS HPLC Column (4.6 × 250 mm) (Bischoff Chromatography, Germany), ellagic acid analysis was carried out. The solvent A and solvent B, or solvent A:solvent B = 80:20, contained 0.5% acetic acid in deionized water and 0.5% acetic acid in acetonitrile, respectively, as the mobile phases. Each and every reagent was HPLC grade. The HPLC was operated at a flow rate of 1.0 ml/min for 50 min. The compounds that were separated were identified using a UV detector that operated at 254 nm.

### Animals Studies

Animal experiments were approved by the Animal Management and Experimental Animal Use Ethics Committee (Registration No. CBF-G-20-2). Six-week-old male Sprague–Dawley rats (*n* = 40) were used for the in vivo assessments and were purchased from Japan SLC, Inc. (Japan). The rats were housed under a controlled temperature (25°C), humidity (50 ± 5%), and a 12 h light/dark cycle. Prior to initiating the experiment, the rats were allowed to adapt to the modified conditions for one week. Rats were weighed and divided into five groups (*n* = 8 per group): normal (N), 60% high-fat diet (HFD) (control; CON), 2% CMC solution (positive control; PC), and HFD + oral JMWE (T1, T2) groups. The HFD + oral JMWE group was further divided into two groups: the T1 group was administered a low concentration of JMWE (1 mg/ml), and the T2 group was administered a high concentration of JMWE (10 mg/ml). HFD was administered to all rats, except for animals in the CON group. At the end of the 12-week period, all rats were fasted for one day, anesthetized with 80 mg/kg tiletamine/zolazepam (Zoletil; Vibrac Laboratories, France), administered intraperitoneally, according to the manufacturer’s instructions, and euthanized via cervical dislocation. The liver and epididymis were harvested, rinsed, weighed, and stored at -70°C until further analysis.

### Tissue Acquisition and Blood Collection

Experimental animals were sacrificed within 24 h of restricting feed containing HFD or normal feed. For analysis, blood samples were collected via retroorbital sinus puncture. The blood was centrifuged, and the supernatant was placed in a tube containing paraffin to prevent clotting. The blood paraffin was centrifuged at 450 g for 20 min at 4°C and stored at -80°C until further analysis. The prepared specimens were tested using a biochemical analyzer for accuracy. The liver and epididymis were harvested, rinsed with PBS, wiped with a paper towel, weighed, frozen in liquid nitrogen, and stored at -80°C until subsequent use.

### Serum Biochemical Analysis

The supernatant separated from the blood was stored on ice for subsequent biochemical analyses. Each supernatant was analyzed to determine levels of HDL-cholesterol, low-density lipoprotein (LDL), total cholesterol, and TG using a biochemical analyzer (Thermo Electron Corp., Finland) and Konelab system reagents.

### Statistical Data Analysis

For multiple comparisons, data were analyzed using Duncan's multiple range test using SPSS (version 18.0; IBM, USA). A statistically significant value was defined as *p* < 0.05. Every piece of data is displayed as mean ± standard deviation (SD).

## Results

### Antioxidant Activities

We assessed the TPC, the reducing power of JMWE, and antioxidative activity using DPPH and ABTS radical scavenging activity. As shown in Table 2, the TPC of JMWE was 133.33 ± 2.60 mg GAE/g. Furthermore, Table 2 illustrates the concentration-dependent increase in DPPH and ABTS radical scavenging activities and reducing power. Notably, at a concentration of 100 μg/ml, the ABTS of JMWE was significantly higher than that of butylated hydroxyanisole (BHA), used as the control. The reducing power values of BHA, AA, and JMWE at a concentration of 100 μg/ml were 0.399, 0.338, and 0.276 μg/mL, respectively.

### Cell Viability

**Fig. 1:**
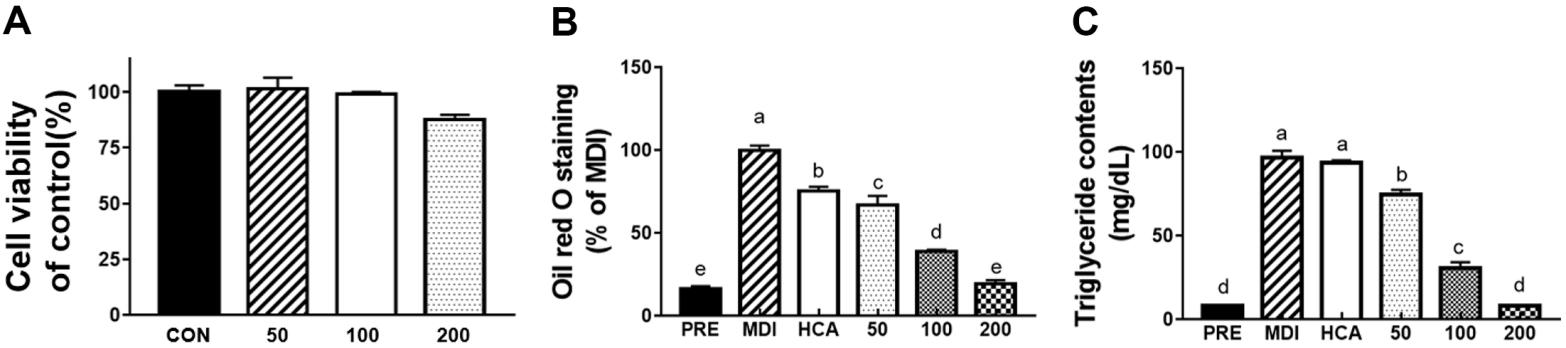
(A) Effect of the JMWE on cell viability in 3T3-L1 cells following treatment with various concentrations (50, 100, and 200 μg/ml) of JMWE for 48 h examined using the CCK-8 assay; (B) Effect of the JMWE on the lipid accumulation in 3T3-L1 cells. Inhibitory effect of JMWE on lipid accumulation in 3T3-L1 adipocyte differentiation using Oil Red O staining. The cells were incubated with MDI solution for 8 days in the presence or absence of JMWE; (C) Effect of JMWE on TG accumulation in 3T3-L1 adipocyte differentiation. Extracellular TG accumulation was determined using a chemical analyzer. Each value denotes the mean ± standard deviation (SD) in triplicate. Means with different letters on the bars indicate significant differences at *p* < 0.05. JMWE, *Juglans mandshurica* Maxim walnut ethanol; TG, triglyceride; PRE, undifferentiated normal control cell; MDI, 0.5 mM 3-isobutyl-1- methylxanthine, 10 μg/ml insulin, and 1 μM dexamethasone.

Based on the cell viability results, JMWE does not affect cell viability below 200 μg/ml (Fig. 1A).

### Effect of JMWE on 3T3-L1 Cell Differentiation

To differentiate 3T3-L1 cells, we used a differentiation medium containing DMEM with IBMX, DEX, and INS to generate lipid droplets. After incubation for 8 days, the differentiated cells were stained using an Oil Red O staining solution. The greater the number of lipid droplets, the more intense the red color of the extraction solution. These results revealed that the MDI extraction solution was red, but the differentiated PRE was colorless. To confirm the anti-obesity effects of JMWE on adipocyte differentiation, the 3T3-L1 cells were treated with 50, 100, and 200 μg/ml of JMWE. After day 8, JMWE markedly and dose-dependently suppressed total lipid accumulation during differentiation. Treatment with 200 μg/ml JMWE reduced total lipid accumulation by over 75% when compared with that in the control (Fig. 1B).

### Inhibitory Effect of JMWE on Extracellular TG Accumulation

A biochemistry analyzer was used to measure the TG contents and analyze the reduction effect of JMWE on the TG content. We observed that 200 μg/ml of JMWE exerted the most potent effect on reducing the TG, according to a concentration-dependent comparison. The results revealed that the TG content of JMWE-treated groups was similar to that of the control group (Fig. 1C).

### JMWE-Mediated Suppression of Adipogenesis-Related mRNA

**Fig. 2:**
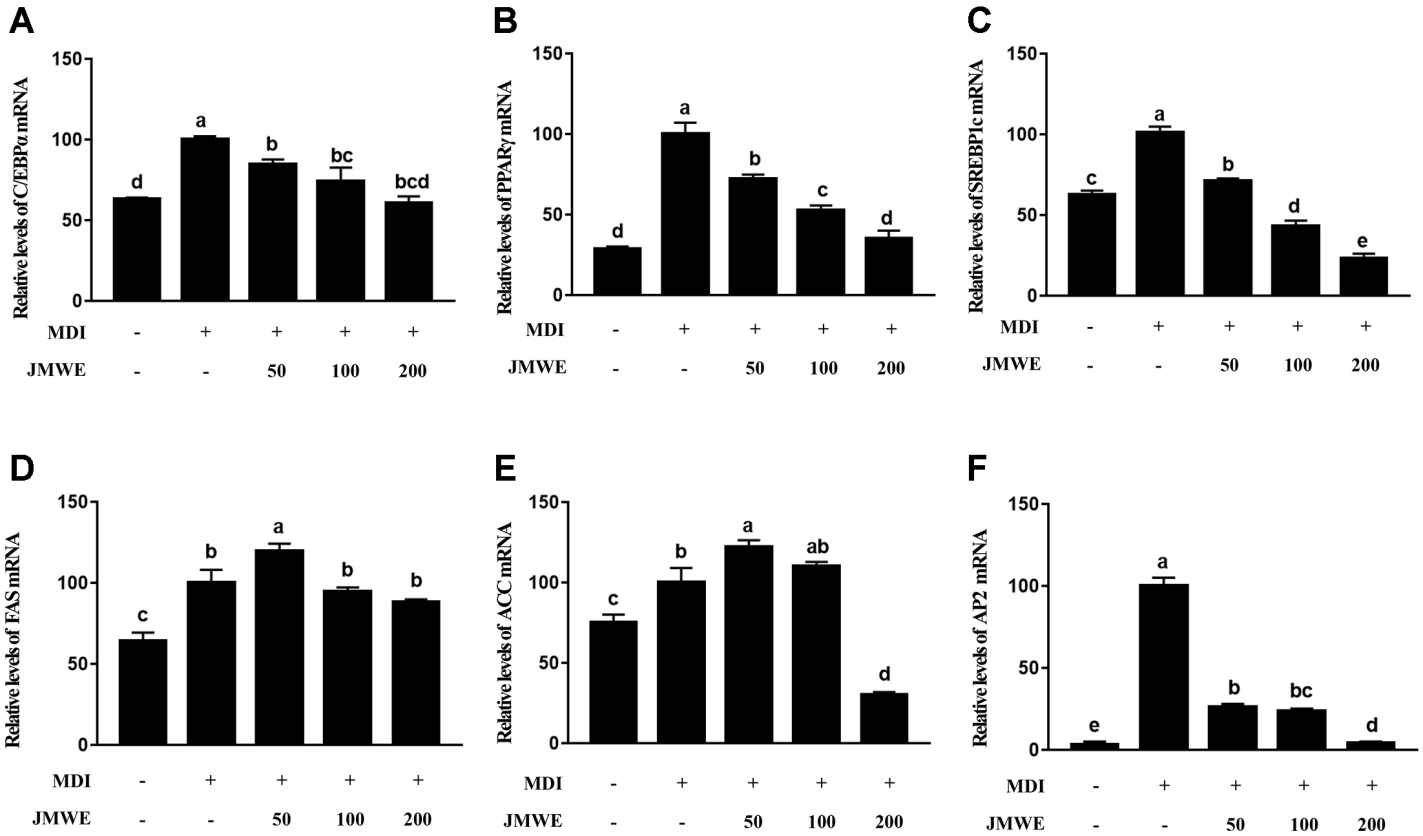
mRNA levels of C/EBPα, PPARγ, SREBP1c, FAS, ACC, and AP2 in 3T3-L1 cells after differentiation for 8 days with JMWE, detected using RT-qPCR. Each value denotes the mean ± standard deviation (SD) in triplicate. Means with different letters on the bars indicate significant differences at *p* < 0.05. ACC, acetyl-CoA carboxylase; C/EBPα, CCAAT-enhancer-binding protein alpha; FAS, fatty acid synthase; SREBP1c, sterol regulatory element-binding protein-1c; JMWE, *Juglans mandshurica* Maxim walnut ethanol; PPARγ, peroxisome proliferator-activated receptor-gamma.

Using a biochemistry analyzer, we detected a reduction in the TG content of 3T3-L1 cells. Treatment with 200 μg/ml JMWE markedly suppressed the expression levels of differentiation-related mRNA, including genes C/EBPα, PPARγ, and SREBP1, when compared with MDI treatment (Fig. 2A-2C).

### JMWE-Mediated Suppression of Lipogenesis-Related mRNA

To confirm whether JMWE could impact fat biosynthesis, we detected mRNA expression levels of FAS and ACC, genes directly related to lipogenesis, in JMWE-treated cells and compared levels with those of MDI-treated cells during differentiation. Treatment with 200 μg/ml JMWE substantially reduced the mRNA expression of FAS and ACC, including AP2 Fig. 2D-2F). These findings corroborate those observed in the concentration-dependent lipid accumulation with Oil Red O staining after JMWE treatment (Fig. 1B).

### JMWE-Mediated Suppression of Adipo- and Lipogenesis-Related Proteins

**Fig. 3:**
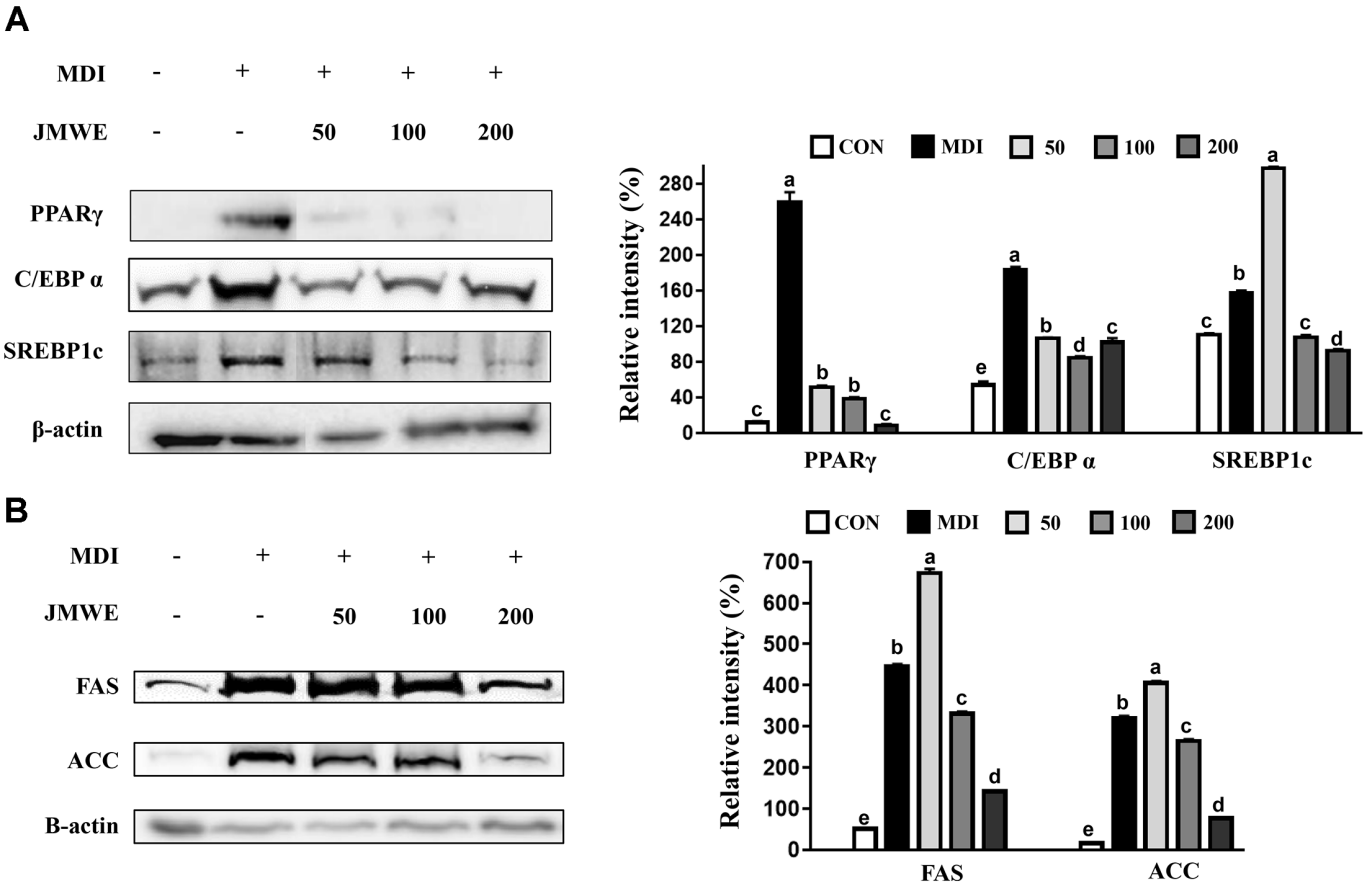
Expression levels of C/EBPα, PPARγ, SREBP1c, FAS, and ACC proteins in 3T3-L1 cells after differentiation for 8 days with JMWE using western blotting. CON, undifferentiated pre-adipocyte; MDI, differentiated adipocyte; 50, 100, and 200 μg/ml, adipocytes treated with 50, 100, and 200 μg/ml of JMWE with MDI. Each value denotes the mean ± standard deviation (SD) in triplicate. Means with letters on the bars denote significant differences at *p* < 0.05. ACC, acetyl-CoA carboxylase; C/EBPα, CCAAT-enhancer-binding protein alpha; FAS, fatty acid synthase; SREBP1c, sterol regulatory element-binding protein-1c; JMWE, *Juglans mandshurica* Maxim walnut ethanol; PPARγ, peroxisome proliferator-activated receptor-gamma.

Fat differentiation can be explained by adipogenesis and lipogenesis. Initially, we confirmed that treatment with 200 μg/ml JMWE could substantially reduce levels of C/EBPα, PPARγ, and SREBP1c proteins during the adipogenesis process. Considering lipogenesis, treatment with JMWE decreased the expression of FAS and ACC, proteins directly involved in fat production, in a concentration-dependent manner (Fig. 3).

### Effect of JMWE Intake on Body and Organ Tissue Weights in an Obese Rat Model

**Fig. 4:**
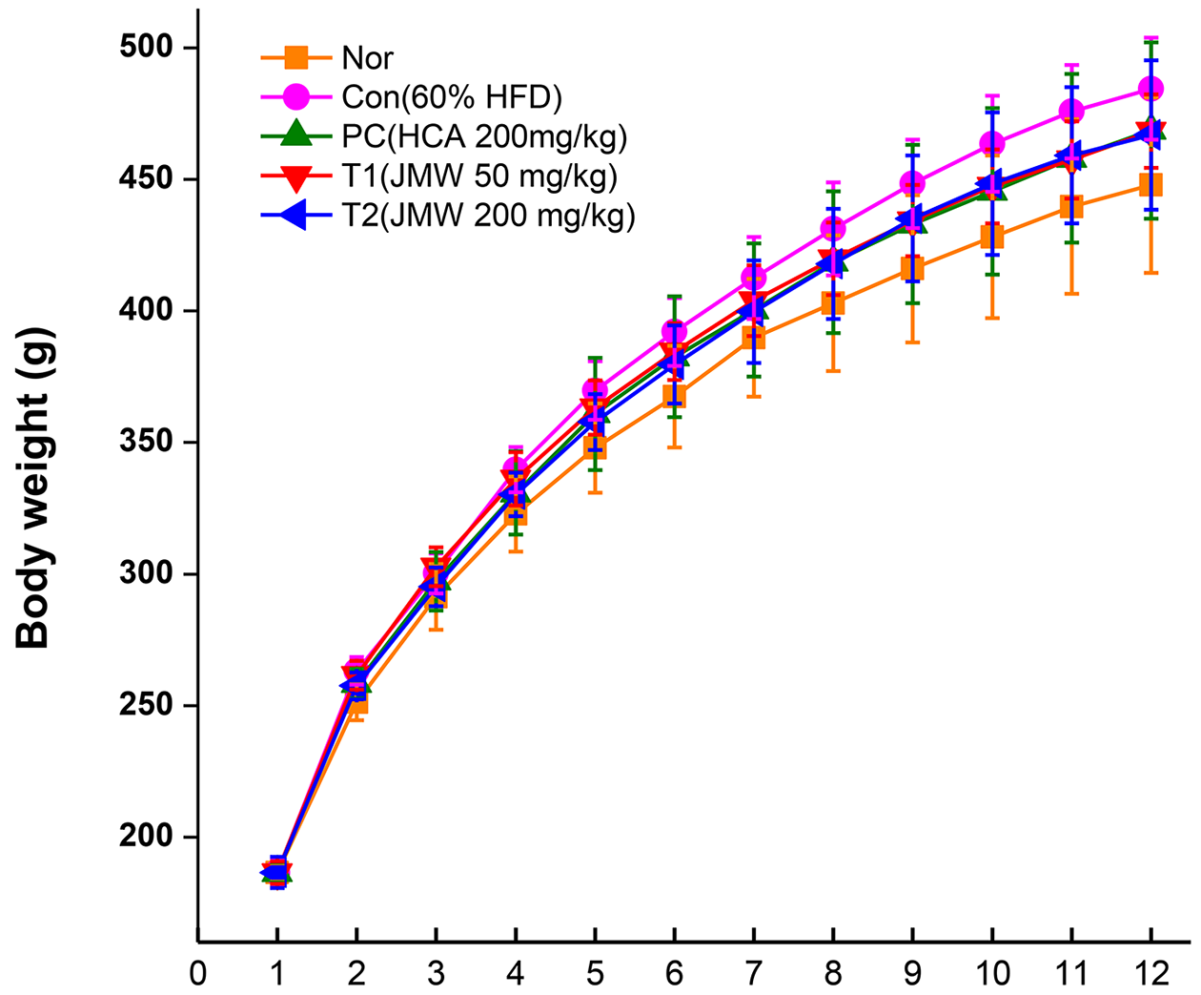
Effect of JMWE on HFD-induced obesity in Sprague–Dawley rats. Body weight measurements were performed thrice weekly. The group that received HFD alone (●) shows steady weight gain, while the HCA group (▲), used as a PC, exhibits body weight loss when compared with the HFD only group; weight loss can be observed in the lowconcentration JMWE (▼) and high-concentration JMWE (◀) groups. HFD, high-fat diet; JMWE, *Juglans mandshurica* Maxim walnut ethanol.

The anti-obesity efficacy of JMWE was assessed using a relevant rat model. The experimental animals were divided into two groups: rats fed normal diets and those fed an HFD (60% fat). The highest body weight (484.5 ± 19.4 g) was observed in the group fed an HFD for 12 weeks. However, treatment with low- and high-concentrations JMWE reduced the body weight by 16.1 and 18.1 g, respectively. Additionally, to confirm the anti-obesity effect of JMWE, we compared the size of the fatty liver and epididymis. The T1 and T2 groups had smaller organ sizes than the control group, suggesting a slow fat accumulation rate in the liver (Fig. 4).

### Effect of JMWE on the Serum and Lipid Profile Levels

The serum lipid profile of obese rats differed significantly (*p* < 0.05) between HFD- and normal diet-fed rats. The T1 and T2 groups exhibited TG concentrations of 31.52 and 28.87 mg/ml, respectively, demonstrating the concentration-dependent reduction in the TG content (Table 3).

### Chemical Composition of JMWE

**Fig. 5:**
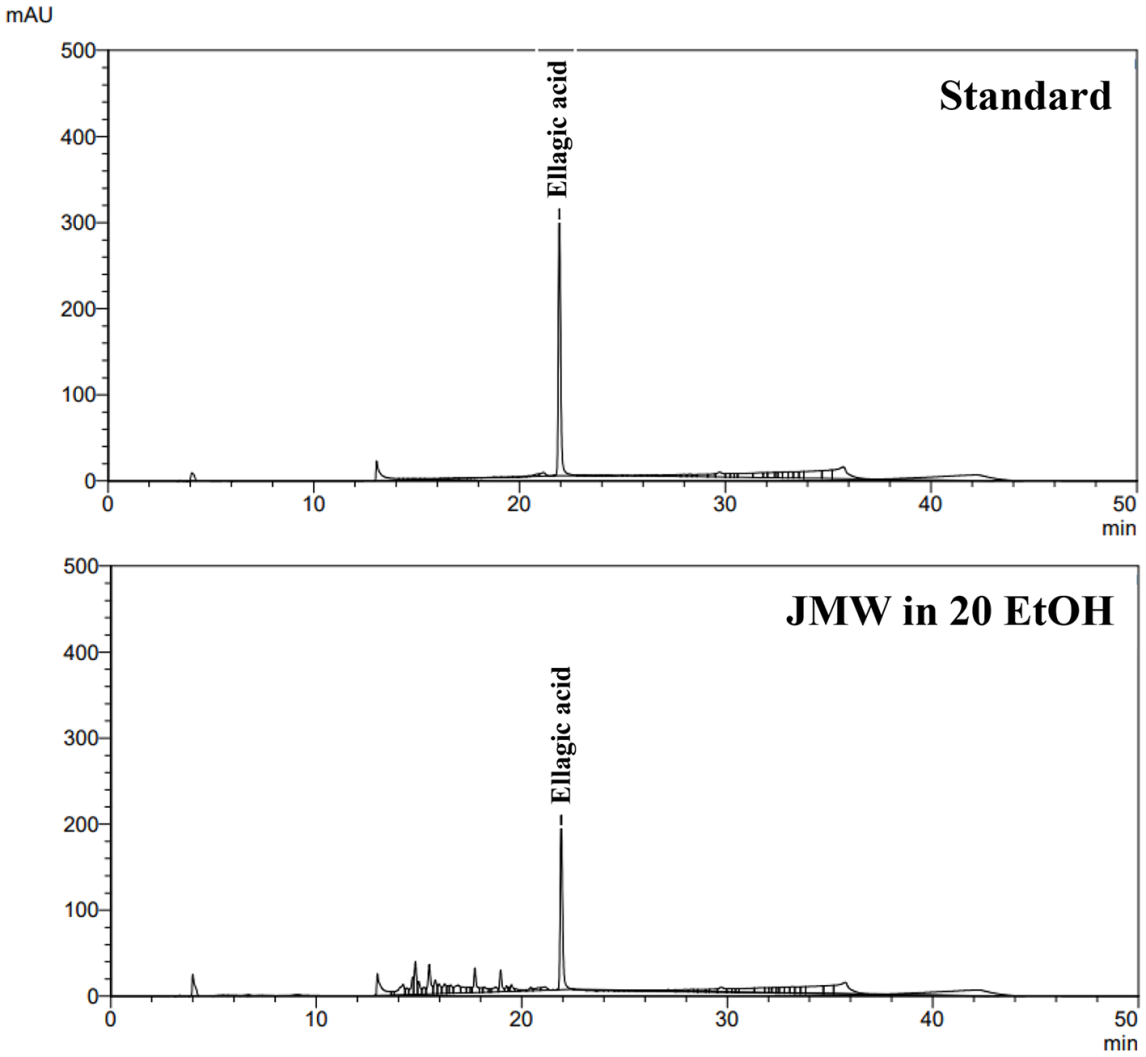
HPLC chromatograms of chemical compounds from JMWE. HPLC, high-performance liquid chromatography; JMWE, *Juglans mandshurica* Maxim walnut ethanol.

HPLC-based quantitative analysis was performed to determine the content of anti-obesity substances present in JMWE (Fig. 5). Ellagic acid was identified as an effective substance present in JMWE, potentially mediating anti-obesity effects. The ellagic acid standard solution was prepared at a concentration of 0.25 mg/ml and subsequently analyzed. The retention time was measured at 21.905 min. The HPLC analysis confirmed the presence of ellagic acid in JMWE at a concentration of 2.93 ± 0.02 mg/g (Fig. 5).

## Discussion

Recent studies have confirmed the presence of various active ingredients in natural products, potentially exerting anti-inflammatory [23], whitening [24], anti-obesity [25], and antioxidant [26-30] properties.

Accordingly, a growing number of individuals prefer consuming natural products as food sources of medicinal compounds rather than the direct consumption of medications owing to the limited side effects [31]. Herein, we aimed to confirm the anti-obesity effect of *J. mandshurica* fruits collected in Korea.

Prior to initiating the current study, extraction was performed using various solvents, including ethanol and methanol, at concentrations of 20, 50, and 70% to select the optimal solvent effective against anti-obesity- We confirmed that the 20% ethanol extract exerted the highest anti-obesity effect. Owing to solvent optimization, the 20% ethanol extract exhibited better anti-obesity efficacy than the 70% methanol extract, which can be applied more widely in various industries.

Accordingly, we next aimed to confirm the anti-obesity and fat decomposition effects of JMWE, a 20% ethanol extract [32, 33]. Ellagic acid has been shown to modulate the formation and differentiation of brown adipose tissue (BAT) [34]. Moreover, ellagic acid can impact anti-obesity by upregulating the rate at which brown adipocytes express PGC-1α and PPARα. In rodent studies, ellagic acid was shown to ameliorate obesity by managing the development and differentiation of white adipose tissue [34]. In addition, we detected mRNA and protein expression rates to confirm their role in the anti-obesity mechanism. Herein, the anti-obesity effect of JMWE containing ellagic acid was confirmed. Additionally, treatment with JMWE dose-dependently reduced the expression of transcriptional regulators related to preadipocytes, mature adipocytes, and lipogenesis, confirming an anti-obesity effect. Moreover, we examine lipid droplet production, lipogenesis [35], and pre-adipocyte-derived inflammation [36-39] in 3T3-L1 cells after treating obese rats with a 20% ethanol extract of JMWE. Based on our findings, JMWE could impact preadipocytes, mature adipocytes, and lipogenesis and suppress cell differentiation.

In preadipocytes, ellagic acid was found to suppress total lipid accumulation by lowering transcriptional regulatory factors such as PPARγ and C/EBPα [40-43].

Furthermore, treatment with JMWE reduced the expression of FAS and ACC, transcriptional regulators involved in TG expression [39, 44, 45]. In addition, in the in vivo experiments in rats, the anti-obesity effects of JMW were based not only on the weight gain results but also on the improved serum profiles.

## Conclusion

In the current study, we determined the effects of JMW on adipogenesis in 3T3-L1 adipocytes. JMWE administration could dose-dependently reduce total lipid accumulation and TGs. Additionally, JMWE suppressed the expression of anti-obesity marker genes at both mRNA and protein levels. Furthermore, JMWE suppressed adipogenesis-related factors C/EBPα, PPARγ, and SREBP1c, as well as FAS and ACC, known to be involved in lipid differentiation. Animal experiments also verified the potential of JMWE for the prevention and treatment of obesity-related diseases. In the future, ellagic acid-containing JMW will contribute not only to the development of healthy functional foods but also to the development of obesity-related drugs through continued research on various active ingredients.

## Figures and Tables

**Table 1:** List of primers used for reverse transcription-quantitative PCR analysis.

Primers	Sequences (5'→3')
C/EBPα	
Forward	CGC AAG AGC CGA GAT AAA GC
Reverse	CAC GGC TCA GCT GTT CCA
PPARγ	
Forward	CGC TGA TGC ACT GCC TAT GA
Reverse	AGA GGT CCA CAG AGC TGA TTC C
SREBP-1c	
Forward	GTT ACT CGA GCC TGC CTT CAG G
Reverse	CAA GCT TTG GAC CTG GGT GTG
ACC	
Forward	GAA TCT CCT GGT GAC AAT GCT TAT T
Reverse	GGT CTT GCT GAG TTG GGT TAG CT
FAS	
Forward	CTG AGA TCC CAG CAC TTC TTG A
Reverse	GCC TCC GAA GCC AAA TGA G
AP2	
Forward	AAC ACC GAG ATT TCC TTC AA
Reverse	TCA CGC CTT TCA TAA CAC AT
β-Actin	
Forward	GTT GGA CCT GAC AGA CTA CCT CA
Reverse	GTT GCC AAT AGT GAT GAC CT

**Table 2:** Antioxidant assays of *Juglans mandshurica* Maxim walnut ethanol (JMWE) extract.

	TPC^1)^ (mg GAE/g)	DPPH (%, μg/ml)				ABTS (%, μg/ml)				Reducing power (Absorbance value)			
		50	100	200	400	50	100	200	400	50	100	200	400
JMWE^2)^	133.33±2.60^3)^	45.63±4.49^d^	64.94±1.14^c^	79.38±1.55^b^	81.25±0.22^a^	57.68±2.06^d^	91.11±1.54^c^	95.69±0.94^b^	99.00≥^a^	0.258±0.00^c^	0.276±0.00^b^	0.311±0.00^a^	0.398±0.00^a^
BHA^4)^		79.86±2.24^b^	89.58±0.10^a^	90.69±0.91^a^	91.92±0.46^a^	57.68±2.06^c^	57.68±2.06^c^	99.00≥^a^	99.00≥^a^	0.360±0.00^c^	0.399±0.00^b^	0.408±0.00^a^	0.425±0.00^a^
AA^4)^		94.67±0.44^b^	94.73±0.00^b^	95.08±0.18^a^	95.26±0.00^a^	99.00≥^a^	99.00≥^a^	99.00≥^a^	99.00≥^a^	0.202±0.00^c^	0.338±0.00^b^	0.399±0.00^a^	0.421±0.00^a^

^1)^TPC: total phenolic contents are expressed as milligrams gallic acid equivalents (GAE).

^2)^JMWE: *Juglans mandshurica* Maxim walnut 20% ethanol extract.

^3)^Data represent the means ± standard deviation (SD) in triplicate.

^4)^Positive controls were used butylated hydroxyanisole (BHA) and ascorbic acid (AA), respectively.

^a-d^: Means with the different superscripts within the same row are significantly different at *p* < 0.05

**Table 3:** Effect of the *Juglans mandshurica* Maxim walnut ethanol (JMWE) extract on serum lipid profiles.

		60% HFD			
	Normal	Control (60% HFD)	Positive control (HCA 200 mg/kg)	T1 (JMWE 50 mg/kg)	T2 (JMWE 200 mg/kg)
HDL (mg/dL)	50.69 ± 8.05	47.26 ± 6.54	48.82 ± 6.19	44.44 ± 6.33	45.01 ± 5.42
LDL (mg/dL)	17.37 ± 3.29	19.42 ± 2.44	20.58 ± 2.23	18.44 ± 2.31	17.85 ± 1.71
Total cholesterol (mg/dL)	50.03 ± 8.72	55.41 ± 5.52	54.11 ± 7.7	50.25 ± 7.44	50.85 ± 5.22
TG (mg/dL)	45.82 ± 10.34	41.73 ± 10.01	32.12 ± 4.47	31.52 ± 5.42	28.87 ± 1.95

HDL, high-density lipoprotein; HFD, high-fat diet; LDL, low-density lipoprotein; TG, triglycerides

Values are expressed as the mean ± standard deviation (SD; *n* = 8 per group). Statistical significance compared with the HFD group (One-way ANOVA followed by Dunnett’s multiple comparison test): *p < 0.05. Statistical significance compared with the control group (One-way ANOVA followed by Dunnett’s multiple comparison test): *p < 0.05.
